# Molecular Investigation of SARS–CoV-2 Proteins and Their Interactions with Antiviral Drugs

**DOI:** 10.3390/v12040445

**Published:** 2020-04-14

**Authors:** Paolo Calligari, Sara Bobone, Giorgio Ricci, Alessio Bocedi

**Affiliations:** Department of Chemical Sciences and Technologies, University of Rome Tor Vergata, Via della Ricerca Scientifica 1, 00133 Rome, Italy; paolo.calligari@uniroma2.it (P.C.); sara.bobone@uniroma2.it (S.B.); riccig@uniroma2.it (G.R.)

**Keywords:** COVID-19, SARS–CoV-2, coronavirus, viral protease, spike protein, antiviral drug, RNA-dependent RNA-polymerase, viral protein *N*, molecular modeling, molecular docking

## Abstract

A new Coronavirus strain, named SARS-CoV-2, suddenly emerged in early December 2019. SARS-CoV-2 resulted in being dramatically infectious, with thousands of people infected. In this scenario, and without effective vaccines available, the importance of an immediate tool to support patients and against viral diffusion becomes evident. In this study, we exploit the molecular docking approach to analyze the affinity between different viral proteins and several inhibitors, originally developed for other viral infections. Our data show that, in some cases, a relevant binding can be detected. These findings support the hypothesis to develop new antiviral agents against COVID-19, on the basis of already established therapies.

## 1. Introduction

In early December 2019, a sudden and dramatic outbreak of a new Coronavirus strain, named SARS–CoV-2, emerged in the continental Chinese area of Wuhan and quickly diffused throughout the country. To date, several cases of viruses were reported in Europe and in the US. The SARS–CoV-2 is strongly infectious, with more than 81,000 infected people and around 3000 deaths reported within three months from the virus appearance (update on 14th of April: more than 1.9 milion of confirmed cases and about 120 thousands deaths reported). The rapid spread of the new virus reveals that the mechanism of diffusion is somehow different from that of *Filoviridae*, (i.e., Ebolavirus) [1]. The viral pathogenesis results in symptoms like fever, acute pneumonia, and, eventually, respiratory failure. The death rate, the long time needed for a full recovery, and the co-morbidity associated with viral infection (i.e., in advanced age or particular life stages, such as pregnancy) result in a major threat to global health [2].

In this scenario, the need for a cure appears to be extremely urgent. The main strategy against the diffusion of viruses is represented by the development of specific vaccines. However, the complexity of viruses and their ability to mutate and adapt to new host organisms belonging to other species has also led to different therapeutic approaches aimed to interfere with the viral life cycle or with processes essential for membrane fusion or replication [3,4]. Furthermore, computational chemistry represents a fundamental tool in the screening of applicable drugs [5]. The computational approach can also represent a tool for physicians and healthcare professionals to operate a first decision on the best therapy to administrate at first instance.

Coronaviruses are single-stranded RNA enveloped viruses [6,7], whose genes encode, among others, for a trimeric structural spike protein, a homodimeric cysteine proteinase [8], an RNA polymerase, and several nonstructural proteins (https://www.ncbi.nlm.nih.gov/nuccore/NC_045512) [9].

From a phylogenetic point of view, the genetic similarities shared by SARS–CoV-2 and the 2002 SARS coronavirus strain (SARS–CoV; they both belong to the so-called beta CoV group) and other bat-isolated coronaviruses strains have already been underlined, with more than 96% gene identity [7,10,11]. The alignment of spike protein and protease showed an analogy in primary sequences of more than 75% [6,10] and 96%, respectively [6,12].

Proteases and spike proteins are targets of choice for inhibition of SARS and MERS [13,14,15,16], and several efforts have been made to develop inhibitors of their activities, by using both virtual screening or experimental methods [5,15].

In this work, we present the results obtained for SARS–CoV-2 spike protein and 3C-like protease by molecular docking of several inhibitors originally developed for antiviral therapy against other viruses, such as the hepatitis C virus (HCV) and the human immunodeficiency virus (HIV). Thirteen proteinase inhibitors from anti-HIV and anti-HCV drugs were investigated, along with three different compounds for spike protein inhibition. Despite the different viral strains for which these drugs were originally developed, we aim to investigate whether they could also have an affinity for the SARS–CoV-2. The use of antivirals that are already available in the therapy against the new virus would be a huge advantage in the fight of this battle, and a first step can eventually arise from bioinformatic analysis, which could provide information about their potential effectiveness. Indeed, our in silico data suggest that an effective interaction occurs for some of the tested molecules.

These results represent a promising starting point for antiviral therapies that are alternative or coadjuvant to the vaccination strategy.

## 2. Materials and Methods

### 2.1. Protein and Antiviral Drugs

#### 2.1.1. Initial Sequences

Protein sequences were obtained from the curated genomic sequence deposited at the Gene database with ID number NC_045512 and were verified against SARS–CoV (ID: NC_004718.3) [17]: the 3C-like protease (ID: YP_009725301.1), the envelope spike protein (ID: YP_009724390.1), the RNA-dependent RNA-polymerase (ID: YP_009725307.1), and the nucleocapsid phosphoprotein (ID: YP_009724397.2) were selected. 

The set of antiviral drugs was taken from the Influenza Research Database (fludb.org). Potential inhibitors of SARS-CoV-2 protease were identified among all the anti-protease drugs in the mentioned database: Asunaprevir (DB11586), Nelfinavir (DB00220), Simeprevir (DB06290), Faldaprevir (DB11808), Indinavir (DB00224), Ritonavir (DB00503), Amprenavir (DB00701), Tipranavir (DB00932), Atazanavir (DB01072), Saquinavir (DB01232), Darunavir (DB01264), Fosamprenavir (DB01319), Lopinavir (DB01601). Furthermore, three molecules were chosen for the spike protein: Umifenovir (DB13609), Enfuvirtide (DB00109), and Pleconaril (DB05105).

#### 2.1.2. Compound Three-Dimensional Structures

The software *OpenBabel* [18] was used to convert the SMILES code of each molecule into a 3D structural file. The three-dimensional structure of Enfuvirtide was taken from the available crystallographic structure (PDB ID 3h00, chain A). 

#### 2.1.3. Protein Structure Prediction: Homology Modelling

The homology model was performed with the iTasser server giving as input the sequences obtained from the SARS–CoV-2 genomic sequence. Model structures were energy minimized before the docking protocol by performing a short in vacuum 500 step steepest-descent optimization of the potential energy using GROMACS tools [19]. 

### 2.2. Docking

Autodock Vina [20] was used to perform molecular docking of the antiviral drugs onto SARS–CoV-2 protease and envelope protein. Regarding the protease, residues 41, 46, 140, 142, 145, 163, 166, 168, 189 were set as flexibles during the binding mode search [21]. About 3C-like protease, the binding box was centered on the coordinates of residue Met165, and its volume fully encompassed the whole binding pocket. For the spike envelope glycoprotein, the box used for the search of binding modes was centered on the position of the center of mass of Val503 side-chain and restrained to the area above the extracellular head of the trimeric protein in the pre-fusion conformation.

## 3. Results

In the following paragraphs, we will analyze and discuss the key properties of putative target proteins from SARS–CoV-2 in comparison with their homologs from SARS–CoV. We will focus in particular on four proteins: the main 3C-like protease, the spike envelope glycoprotein, the RNA-dependent RNA-polymerase (RdRp), the Nucleocapsid protein.

### 3.1. 3C-Like Protease

#### 3.1.1. Structural Analysis

The 3C-like protein is the main protease of SARS-CoV-2. It plays a fundamental role in RNA translation and, thus, as already underlined, is essential for viral replication [12]. In the mature form, it is found as a dimer. Each monomer is formed by three structural pseudo-domains: domain I (residues 8–101), domain II (residues 102–184), which share an antiparallel β-barrel structure, and domain III (residues 201–303), which contains a five-fold antiparallel α-helix cluster [22,23]. The binding site for substrates is located in a cleft region between domains I and II, and the catalytic region is formed by the dyad His41-Cys145 that is highly conserved among the coronavirus proteases and is also reminiscent of the trypsin-like serine proteases [22]. 

**Figure 1:**
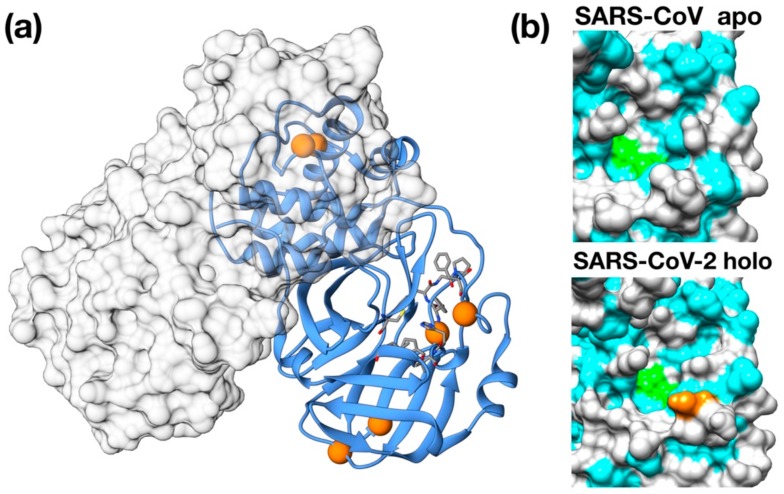
Structural features of 3C-like protease from SARS–CoV-2. (**a**) Homology model structure with chain A shown as ribbons and chain B as molecular surface. Residues mutated with respect to the SARS–CoV homologue are shown as spheres. Active site residues are shown as stick. (**b**) Surface representation of the catalytic site of SARS–CoV Main protease (PDB ID: 5B6O) and of the crystallographic structure of inhibitor-bound SARS–CoV-2 3C-like protease (PDB ID: 6LU7). Hydrophobic residues are shown in cyan. Catalytic residues (His41, Cys145) are shown in green. Ala46Ser mutation is shown in orange on the SARS–CoV-2 structure.

Importantly, 3CPro-19 from SARS–CoV-2 shares a high similarity with its SARS–CoV homolog [24], and only very few residues are substituted with respect to the SARS counterpart: Thr35Val, Ala46Ser, Ser94Ala, Lys180Asn, Ala267Ser, Thr285Ala. Most of these residues are distant from the protease active site and are unlikely related to selectivity against this protease (Figure 1a). Nonetheless, two of these mutations, Lys180Asn and Ala46Ser, are located in the deep hydrophobic pocket below the active site and in the loop region flanking the entrance of the active site. Although in the available crystallographic structure, Lys180Asn results to be located too far to directly contribute to ligand binding, its presence extends the hydrophobic inner region. Conversely, the Ser46 seems to be relatively distant from the His41 active site (11 Å) and may have a role in ligand recruitment (Figure 1b). 

#### 3.1.2. Docking

**Figure 2:**
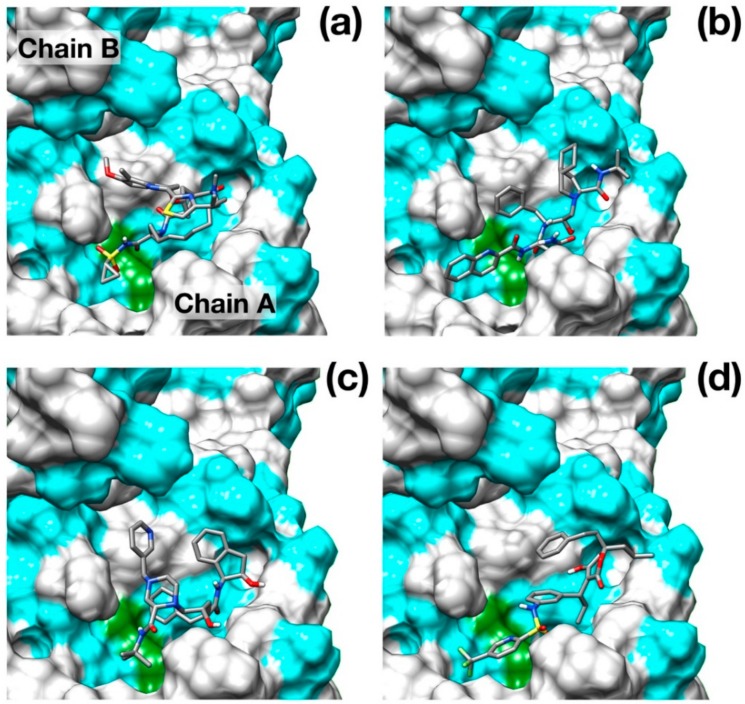
Binding poses of the best scoring docked inhibitors. (**a**) Simeprevir; (**b**) Saquinavir; (**c**) Indinavir; (**d**) Tipranavir. Hydrophobic residues (Ala, Phe, Leu, Ile, Pro, Tyr, Val, Met, Trp, Gly) are colored in cyan. Catalytic residues are colored in green. N, O, C and S atoms are colored in blue, red, gray and yellow respectively.

Although a crystallographic structure of 3Clike protease of SARS-CoV-2 in complex with a peptide-like inhibitor (PDB id: 6LU7) was made very recently available in the Protein Data Bank, this structure clearly shows a closed binding pocket around the inhibitor. While very useful to identify the residues involved in the inhibitory action, this configuration is not very well suited for molecular docking as it may limit the effectiveness of the pose searching methods. For this reason, we preferred to model the three-dimensional structure of the protease using a homology modeling protocol, excluding the complexed covid-19 protease among the target structures. The structure obtained from the iTasser server showed a very good alignment score (TM-score 0.993) against the apo structure of SARS–CoV main protease (PDB ID: 5B6O). Interestingly, the root-mean-squared deviation (RMSD) of the model structure from the available crystallographic structure SARS–CoV-2 protease is as low as 1.3 Å, and it is mostly due to differences in the binding pocket and loop conformations. The main results obtained from the docking protocol are shown in Table 1. Protease drug recognitions are usually driven by hydrophobic interactions [24,25], and Figure 2 clearly shows how all the four best scoring ligands indeed thoroughly fill the hydrophobic pockets that flank the catalytic dyad.

Surprisingly, the well-known HCV NS3/4A protease inhibitor Simeprevir [26] was identified as the best scoring drug with a significant difference with respect to some of the most promising inhibitors of SARS–CoV-2 protease identified, such as Lopinavir [27] and Nelfinavir [28]. 

**Figure 3:**
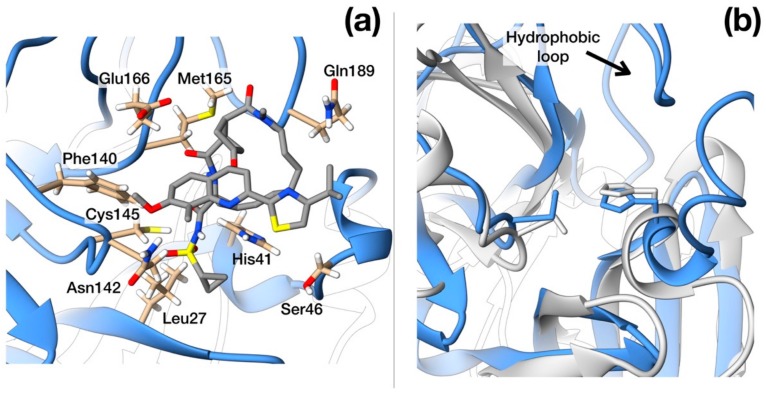
Structural details of SARS–CoV-2 protease. (**a**) Ribbon representation of protease active site with side-chains of residues that interact with Simeprevir shown in full atom details. (**b**) Ribbon representation of the crystallographic structures of SARS–CoV-2 (blue, PDB ID: 6LU7) and HCV protease (light gray, PDB ID: 3KEE) superimposed. The hydrophobic loop Phe181–Phe185 of SARS-CoV-2 protease is evident on the upper side and is absent in HCV homologue. N, O, C and S atoms are colored in blue, red, gray and yellow respectively.

Simeprevir fully fills two hydrophobic pockets flanking the catalytic dyad His41-Cys145: the macrocyclic compound fits in the pocket formed by residues in the range Phe181–Phe185, while the acyl sulfonamide cyclopropyl moiety points towards the region encompassing Leu27, Pro39, and Val42. This pose induces the opening of the catalytic dyad. Indeed, His41-Cys145 changes from 3 Å, in the apo configuration, to 7.3 Å in the inhibited form. Simeprevir binding to SARS–CoV-2 protease is also sustained by three hydrogen bonds that anchor the ligand to the binding pocket: two of them link the amide backbone of Glu166 and Gly143 to the carbonyl *O* in the macrocycle and to the acyl sulfonamide cyclopropyl moiety, respectively. A third hydrogen bond is formed between the *N* of the Asn142 side chain and the ether group. A detailed structural representation of the interacting residues is shown in Figure 3a. These findings may appear rather surprising, as the HCV main protease shares very low similarity with the SARS–CoV-2 homolog (7.5%). Nonetheless, the two proteases share a very similar topology of the active site except for the hydrophobic loop Phe181–Phe185 which is structurally absent in the HCV protease (Figure 3b). 

### 3.2. Envelope Glycoprotein

The so-called spike protein (or S protein) of SARS–CoV-2 is present within the envelope as a homotrimer, like in the case of other enveloped viruses. Each monomer features an ectodomain, a transmembrane domain, and a cytoplasmic tail. Inhibitors are typically designed to interact with the upper part of the protein, to interfere with the host membrane binding, and with the fusogenic activity of the protein. 

#### 3.2.1. Structural Features

Several experimentally obtained structures for the multimeric conformation of SARS–CoV spike protein are available (PDB: 5WRG, 5 × 58, 5XLR). Homology model structure of SARS–CoV-2 spike protein was obtained from the iTasser server. Global pairwise sequence alignment indicated that SARS–CoV-2 spike protein shares about 76% of its primary sequence with its SARS–CoV homolog with an overall similarity of 87%. These few differences project into a slightly different negative/positive residue ratio (115/99 in SARS–CoV and 110/103 in SARS–CoV-2) and a slightly less acidic pI (5.6 for SARS–CoV, 6.2 for SARS–CoV-2). 

Interestingly, while this work was in progress, the electron microscopy structure of the SARS–CoV-2 spike protein in its oligomeric pre-fusion inactive structure with a single receptor-binding domain up was made available by another group (PDB id: 6VSB) [29]. Unfortunately, a thorough direct comparison of our model structure with these experimental data is impaired by the different global configuration of the two trimeric structures (“down”-inactive vs. “up”-active conformations). However, as the experimental structure shows only one monomer in the “up”-state, a comparison can be performed in a monomer-based manner. The RMSD between “down”-state monomers in the two structures is as low as 1.17 Å for residues with the same secondary structure assignments and 6 Å overall. It is worth noting here that in the pre-fusion state, the “down” inactive conformation is significantly more stable than the “up”-conformations [30,31,32]. These findings confirm the quality of the homology model and its suitability for docking studies.

**Figure 4:**
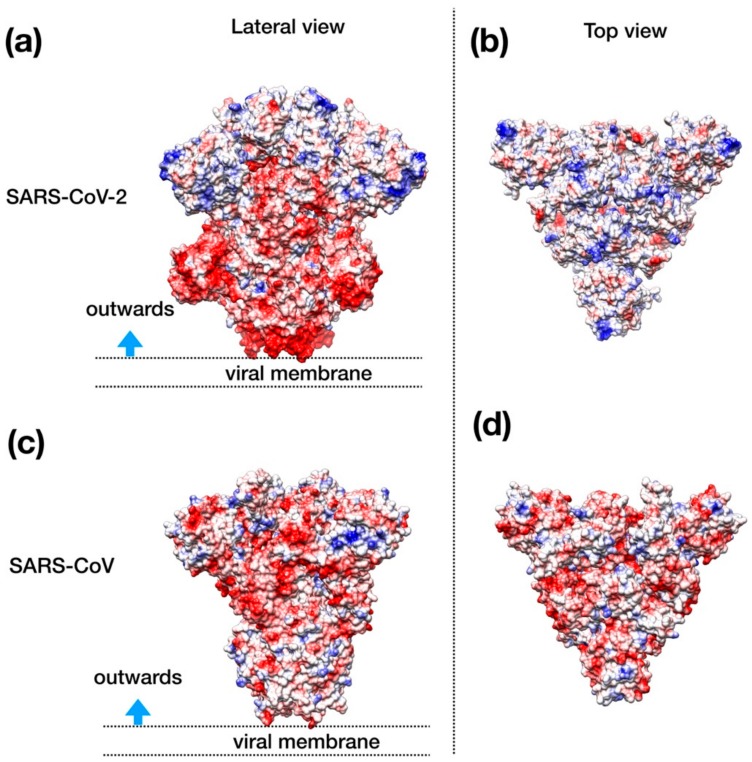
Structural representation of the spike trimer from SARS–CoV-2. (**a**) Molecular surface of the trimer structure, colored according to the local values of the electrostatic potential. The color palette ranges from −10 kcal/(mol·e) (red) to +10 kcal/(mol·e) (blue). (**b**) Top view of the same representation as in panel A. (**c**) Spike envelope protein from SARS-CoV. Same lateral view as in panel (**a**). (**d**) Top view of the same representation as in panel (**c**).

Visual inspection of the electrostatic potential of the “down”-inactive conformation of SARS–CoV-2 spike protein (Figure 4a,b) shows that while the inner part of the protein that is proximal to the viral envelope is negatively charged, the protein head is clearly characterized by a positive electrostatic potential. This picture is rather different from one from SARS–CoV (Figure 4c,d) and, thus, it suggests an important property to which tailor future inhibitors.

#### 3.2.2. Docking

The docking of three different inhibitors was performed on the spike protein. As the inhibitors are expected to block the membrane binding, we limited the docking search region to the area around the protein head in its pre-fusion “down” configuration. Moreover, due to the large size of the protein and to limit the computational cost, we performed a rigid docking of the inhibitors against the target multimeric protein. 

Autodock Vina was used to identify the best binding pose of three different drugs: Umifenovir (DB13609), Enfuvirtide (DB00109), and Pleconaril (DB05105). The results are shown in Table 2. 

Interestingly, upon binding, Enfuvirtide interacts with spike protein (whose cap is mainly positively charged) through its negatively charged amino acids (several glutamate residues). 

**Figure 5:**
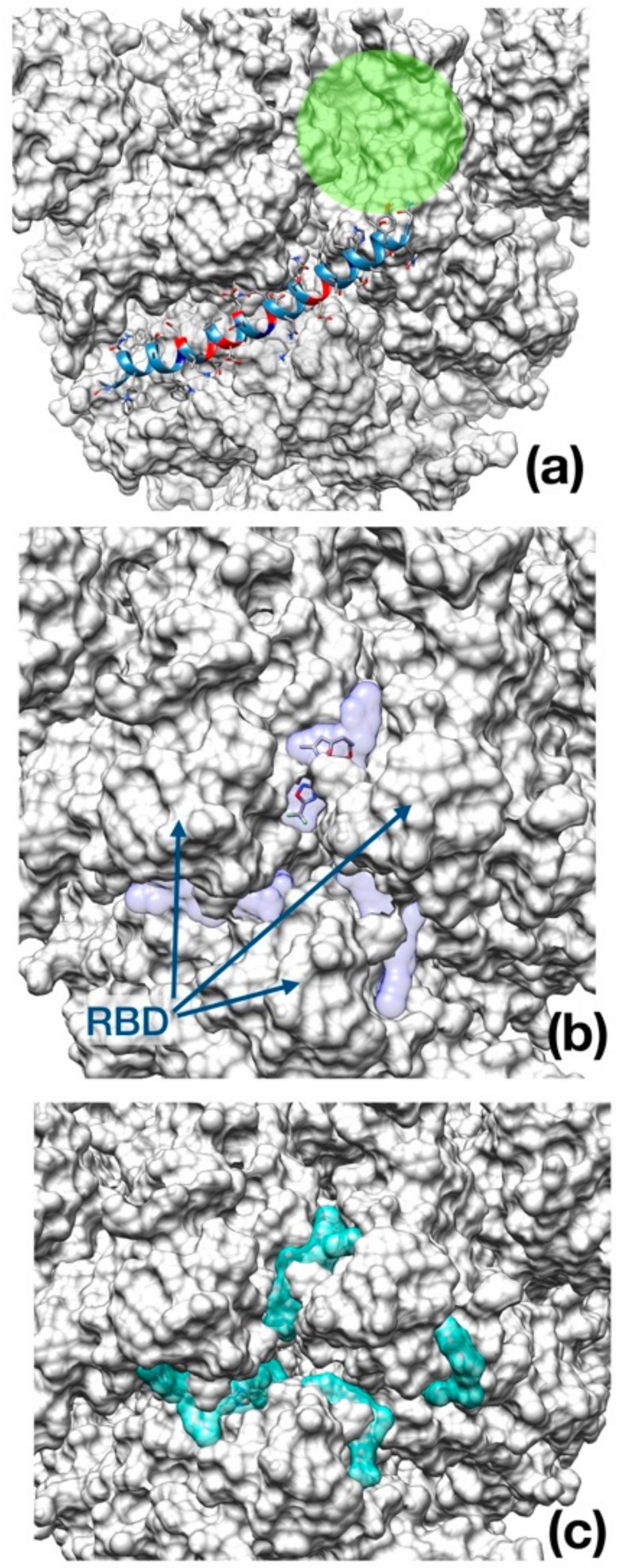
Best binding pose found with AutoDock Vina docking algorithm. (**a**) Enfuvirtide (shown in ribbon representation); (**b**) Umifenovir; (**c**) Pleconaril. The channel within the trimeric cap, is reported as a green circle. In panel (**b**) and (**c**), the transparent surface area indicates the localization of each inhibitor in the best five docking poses for Umifenovir (purple) and Pleconavir (green). The receptor-binding domains (RBD) are indicated by arrows in the middle panel as a reference.

As opposed to Enfuvirtide (Figure 5a), which inhibits the viral spike protein action upon host membrane by interposing itself and avoiding contact between the protein and the cellular membrane, the other two drugs docked at the entrance of the central channel pore formed by the trimeric structure (Figure 5b,c).

### 3.3. RNA-Dependent RNA Polymerase

Viral polymerases share seven highly conserved motifs that are essentially involved in the nucleotide-binding and catalysis of RNA replication. They are huge, complex structures, which act in association with several non-structural proteins [33]. 

#### Structural Properties

**Figure 6:**
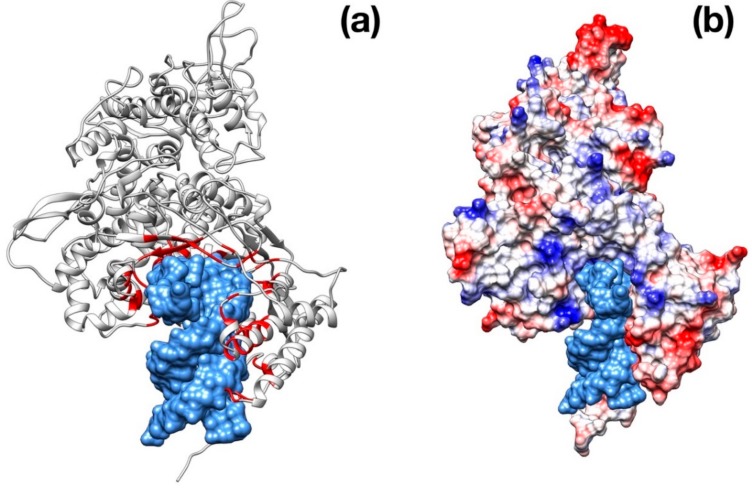
Structural representation of the RdRp monomer from SARS–CoV-2. (**a**) Superposition with the structure poliovirus RdRp in complex with RNA primer (PDB id: 3OL6, only the nucleotide fragment is shown for clarity). The residues in contact with RNA primer are colored in red. (**b**) Same as in panel (**a**) but with SARS–CoV-2 RdRp molecular surface colored according to the electrostatic potential.

Global pairwise sequence between SARS–CoV-2 RdRp and its SARS–CoV counterpart shows a large sequence identity shared by the two proteins (94%) with also a very high similarity (96%). The analysis of RdRp and RNA primer complex displays that the RNA-interacting residues are conserved between SARS–CoV and SARS–CoV-2 (in red in Figure 6a).

### 3.4. Nucleocapsid Protein

The viral nucleocapsid protein (*N* protein) interacts with the viral genome and forms the ribonucleoprotein core. It is now well-known that this protein plays different roles in the viral infection, as it is involved in the synthesis of viral RNA, in the transcription of genomic RNA, and in the translation of viral proteins [34].

**Figure 7:**
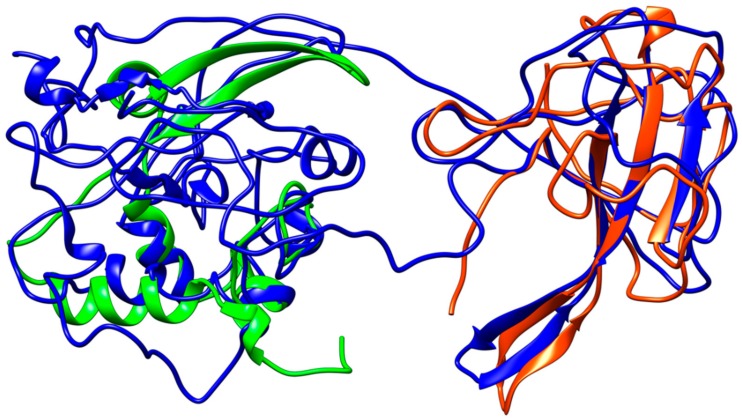
Structural representation of the homology model for Nucleocapsid monomer from SARS–CoV-2 shown in blue and superpose with the structure of the N-terminal fragment from IBV coronavirus homologue (colored in green, PDB ID: 2BXX) and with the dimerization domain of SARS RdRp (in red, PDB ID: 2GIB).

Coronavirus nucleocapsid proteins share a general structural pattern composed of three distinct domains: an N-terminal domain (~130 residues), which is suggested to bind RNA, a central domain (~120 residues), which is also predicted to recognize RNA, and finally the C-terminal domain, which drives the protein dimerization. Although a complete structure of the nucleocapsid protein is not available for SARS–CoV, a local structural comparison can be performed by structure superposition of SARS–CoV-2 model structure with N-terminal and central domain in the SARS counterparts (Figure 7). Sequence pairwise alignment of SARS–CoV-2 *N* protein against its SARS–CoV homolog shows a very high identity in primary structure (91%), with an overall similarity of 94%.

## 4. Discussion 

The modeling and superimposition of protease structure with the homologous SARS virus protease show very little differences, although pivotal for the function. The few residues different in SARS–CoV-2 protease assimilate the protein to a sort of site-directed mutant of SARS protease (Figure 1). The spike protein structure shows an upper, trimeric cap supported by a stem domain, with remarkable differences in terms of charge, with a mainly positive ectodomain and a strongly negative stem domain (Figure 4). 

In this respect, stimulated by the important results in clinical trials of patients treated with antiretroviral agents [35,36], we extended the investigation by a computational inspection of many different drugs, and we chose a selection of most common antiviral drugs to display the probable molecular reasons of their efficacy in combined antiviral therapy. The drugs examined include the HIV-1 protease inhibitors, mostly used in combination with other antiviral drugs in the treatment of HIV in both adults and children. Moreover, other antiviral drugs included in our screening show activity against the hepatitis C virus and good oral bioavailability.

One of the antiviral drugs tested against SARS-CoV-2 envelope protein is Enfuvirtide, an HIV fusion inhibitor used in combination therapy for the treatment of HIV-1 infection [37]. The second molecule tested is Umifenovir, an antiviral agent with activity against Influenza A- and B-viruses [38]. The last one is Pleconaril [39], used for prevention of asthma exacerbations and common cold symptoms in asthmatic subjects exposed to respiratory infections; Pleconaril is orally bioavailable and active against *Picornaviridae* viruses.

The docking shows that 5 out of the 13 protease inhibitors are reported with a high-score in thermodynamic terms of a protein–drug complex [20] (Table 1), and may be selected as the future core of new antiviral drugs specific for SARS–CoV-2 protease. 

Intriguingly, the docking of the spike protein shows interesting results for all the three drugs tested. Notably, Enfuvirtide is placed at the interface of two monomers; in this position, the molecule could impair the transition between closed and open state (Figure 4). 

Finally, it is worth mentioning that, currently, some of the drugs reported in Table 1 are used with satisfying results in therapies for the treatment of SARS–CoV-2.

The RNA-dependent RNA-polymerase (RdRp) is one of the most important proteins involved in the genetic information replication and transmission of the coronavirus family viruses. The function is accomplished by a multimeric complex of RdRp with non-structural proteins favoring a sort of “wrapping-and-scrolling” of the viral genome. The RdRp is very similar to the homologous of SARS (Figure 6).

The *N*-protein promotes the package of viral genome into a helical ribonucleocapsid and virion assembly, also interacting with membrane protein. Consequently, *N*-protein plays an important role in viral RNA transcription, as well as viral replication. The dimerization domains of SARS–CoV and SARS–CoV-2 share a high similarity (Figure 7).

The differences at protein structure level with the SARS, the closest phylogenetic virus, are intriguing and contribute to characterize the peculiarity of SARS-CoV-2 from coronavirus and influenza virus belonging to an old-scenario of virology taxonomy.

It is worth mentioning that several other groups are working on antiviral-based strategies to fight the new, big threat represented by SARS–CoV2; in some cases, patients were treated with some of the molecules chosen for our studies, i.e., Ritonavir, Lopinavir, Umifenovir [27,40,41,42]: these data strongly support and corroborate the results of our work.

## 5. Conclusions

Until now, the exacerbation of COVID-19 diffusion has stimulated the researchers and medical personnel to find a solution for the treatment compatible with the arsenal of antiviral drugs currently available.

At the same time in which we are closing the present manuscript, the numbers are more than 81,000 confirmed cases, around 3000 deaths, and a sensible increase of recovered people (30,000) according to the Coronavirus COVID-19 Global Cases by Johns Hopkins CSSE interactive map (updated on 14th of April with more than 1.9 milion of confirmed cases and about 120 thousands deaths reported). The first line strategy of isolation, dramatic quarantine measurements, and clinical treatments may be a reasonable positive warrant to limit the spread of the virus. 

The four proteins taken into account are interesting molecular targets for the medicinal chemistry, mainly the protease and the envelope (or spike) protein. 

The antiviral drug therapy is an effective first-stage approach for treating COVID-19 infections, and the present work could contribute to suggest to physicians the choice of the combination of antivirals to administer to patients alongside the hospitalization. Obviously, our knowledge in terms of the general organization of the structure of the virus particle is limited now, but proteins like protease and spike are the primary molecular targets for drug discovery and development. In the near future, the availability of X-ray crystal structures of the majority of proteins will allow a more exhaustive docking calculation on different classes of drugs (e.g., antiparasitic drugs, peptides). The structural and computational studies and analysis are not subordinate but helpful for the comprehension of virus molecular elements and the right way to help the scientific community during the long process leading to developing a vaccine.

## Figures and Tables

**Table 1:** Results from molecular docking of thirteen different antiviral protease inhibitors. The second column indicates the original viral target of each compound (hepatitis C virus (HCV), human immunodeficiency virus (HIV)).

Inhibitor	Viral Target	Drugbank ID	Vina Scoring (kcal/mol)
Simeprevir	HCV	DB06290	−10.0
Saquinavir	HIV	DB01232	−9.3
Indinavir	HIV	DB00224	−8.7
Tipranavir	HIV	DB00932	−8.6
Faldaprevir	HCV	DB11808	−8.4
Ritonavir	HIV	DB00503	−8.1
Lopinavir	HIV	DB01601	−8.1
Asunaprevir	HCV	DB11586	−8.1
Atazanavir	HIV	DB01072	−8.0
Nelfinavir	HIV	DB00220	−7.9
Amprenavir	HIV	DB00701	−7.7
Darunavir	HIV	DB01264	−7.6
Fosamprenavir	HIV	DB01319	−7.2

**Table 2:** Results from molecular docking with Autodock Vina.

Inhibitor	Drugbank ID	Vina Scoring (kcal/mol)
Umifenovir	DB13609	−7.7
Pleconaril	DB05105	−7.1
Enfuvirtide	DB00109	−5.9
